# Neuropsychiatric Lupus Erythematosus: Future Directions and Challenges; a Systematic Review and Survey

**DOI:** 10.6061/clinics/2020/e1515

**Published:** 2020-04-13

**Authors:** Yongwen Zhang, Huanhuan Han, Lanfang Chu

**Affiliations:** I Department of Endocrinology and Rheumatology, Nanjing Integrated Traditional Chinese and Western Medicine Hospital Affiliated with Nanjing University of Chinese Medicine, Nanjing 210014, China; II Department of Integrated Traditional Chinese and Western Medicine, General Hospital of Eastern Military Area, Nanjing 210012, China.

**Keywords:** Neuropsychiatric Lupus Erythematosus, Diagnostic Criteria, Management

## Abstract

This study aimed to systematically review neuropsychiatric lupus erythematosus (NPSLE) and establish a simplified diagnostic criterion for NPSLE.

Publications from 1994 to 2018 in the database (Wanfang data (http://www.wanfangdata.com.cn/index.html) and China National Knowledge Internet (http://www.cnki.net)) were included. In total, 284 original case reports and 24 unpublished cases were collected, and clinical parameters were analyzed. An attempt was made to develop a set of simplified diagnostic criteria for NPSLE based on cases described in the survey and literature; moreover, and pathophysiology and management guidelines were studied.

The incidence rate of NPSLE was estimated to be 12.4% of SLE patients in China. A total of 408 NPSLE patients had 652 NP events, of which 91.2% affected the central nervous system and 8.8% affected the peripheral nervous system. Five signs (manifestations, disease activity, antibodies, thrombosis, and skin lesions) showed that negative and positive predictive values were more than 70%, included in the diagnostic criteria. The specificity, accuracy, and positive predictive value (PPV) of the revised diagnostic criteria were significantly higher than those of the American College of Rheumatology (ACR) criteria (χ2=13.642, 15.591, 65.010, *p*<0.001). The area under the curve (AUC) for revised diagnostic criteria was 0.962 (standard error=0.015, 95% confidence intervals [CI] =0.933–0.990), while the AUC for the ACR criteria was 0.900 (standard error=0.024, 95% CI=0.853–0.946). The AUC for the revised diagnostic criteria was different from that for the ACR criteria (Z=2.19, *p*<0.05).

Understanding the pathophysiologic mechanisms leading to NPSLE is essential for the evaluation and design of effective interventions. The set of diagnostic criteria proposed here represents a simplified, reliable, and cost-effective approach used to diagnose NPSLE. The revised diagnostic criteria may improve the accuracy rate for diagnosing NPSLE compared to the ACR criteria.

## INTRODUCTION

Systemic lupus erythematosus (SLE) affects the nervous system, causing a variety of manifestations, including those of the central nervous system (CNS) and peripheral nervous system (PNS). The neuropsychiatric lupus erythematosus (NPSLE) is a severe complication of SLE. NPSLE is characterized by a variety of neurological manifestations; its diagnosis has been a formidable challenge for rheumatologists 1. NPSLE can present with numerous symptoms that are frequently overlooked despite being associated with increased mortality and morbidity 2. Because of the clinical heterogeneity of NPSLE, and because we lack evidence-based treatment and etiological insight, clinical management is complex. Obviously, NPSLE does not occur due to a single pathophysiological mechanism, and mechanisms may vary according to the pathoanatomic localization of NPSLE—CNS, PNS, and vascular.

The American College of Rheumatology (ACR) has identified 19 neuropsychiatric syndromes in SLE 3, which can be divided into CNS and PNS manifestations. Although this classification includes manifestations without clear physiological and pathological mechanisms, it helps rheumatologists identify neurological involvement. The incidence of NPSLE was also thought to have decreased as a result of the improved outpatient detection and treatment of SLE. There was no epidemiological data on NPSLE in China. Moreover, to the best of our knowledge, no revised diagnostic criteria have been reported. This review outlines our clinical studies on NPSLE for approximately 20 years, based on the evidence from literature reviews and surveys, recommendations for the diagnostic criteria and management of NPSLE were established. It is hoped that this effort will be useful to rheumatologists for the diagnosis and treatment of patients with NPSLE.

### Definition of NPSLE

NPSLE 4 is a group of neurologic syndromes of the CNS and PNS and/or of psychiatric syndromes observed in patients with SLE in which other causes are excluded by laboratory, clinical, neuropsychological, and neuroimaging tests 5. NPSLE is a general term used to describe the wide range of SLE-related complications in the CNS and PNS. This syndrome is different from other aspects of SLE because it can occur without any serological changes. Focal disease in the form of focal seizures or stroke is caused by lupus-related coagulopathies 6. However, diffuse disease varies considerably among patients; symptoms include memory impairment, anxiety, general cognitive decline, and depression 7. NPSLE can exhibit manifestations similar to those of hormonal disturbances, metabolic, posterior reversible encephalopathy syndrome, thrombotic thrombocytopenic purpura (TTP), side effects of medications (especially corticosteroids), and infectious.

### Epidemiology of NPSLE

**Figure 1:**
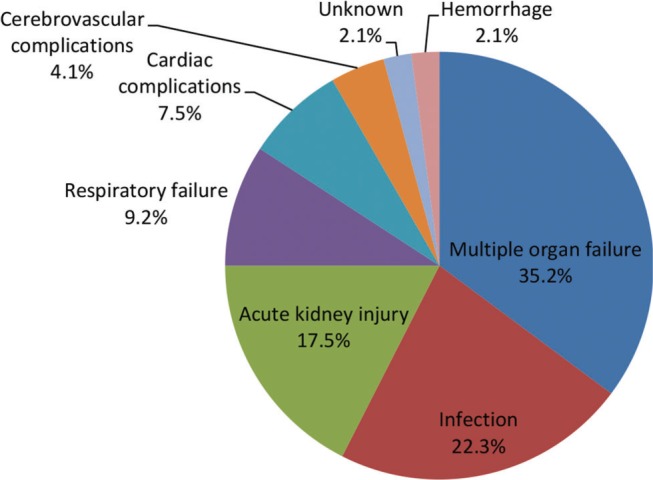
Causes of death in Chinese patients with neuropsychiatric lupus erythematosus.

The reported prevalence of NPSLE ranges from 6% to 91%; this variation is partly due to differences in research methods, such as screening methods, follow-up duration, design types, lack of specificity of many NP manifestations, and heterogeneous procedures of assessments 8,9. A specific study on the prevalence of NPSLE has not yet been performed in China. Thus, the author attempted to investigate NPSLE nationwide. Publications from 1994 to 2018 in the database (Wanfang data (http://www.wanfangdata.com.cn/index.html) and China National Knowledge Internet (http://www.cnki.net)) were included, using the terms “neuropsychiatric lupus erythematosus”, “neuropsychiatric lupus”, “systemic lupus erythematosus AND nervous system”, “systemic lupus erythematosus AND neuropsychiatric”, the exclusion criteria were incomplete clinical data and unclear diagnosis. In total, 284 original case reports and 24 unpublished cases were collected, and the clinical parameters were analyzed. The incidence rate of NPSLE was estimated to be 12.4% of SLE patients in China, accounting for 10.2% of hospitalized patients with SLE. The number of patients with NPSLE managed by rheumatism departments has gradually increased in the last seven years (2011–2018), but has decreased in emergency units and neurology departments. This may be partly due to increased awareness of NPSLE because research activities have often been presented at meetings. Patients with a history of or concurrent NPSLE, APL antibodies, skin lesions, as well as those with generalized disease activity are at a higher risk of developing NPSLE. The most common causes of death were multiple organ failure (MOF, 35.2%), infection (22.3%), and acute kidney injury (AKI, 17.5%) (Figure 1). Multiple logistic regression indicated that pneumonia (OR=3.831; *p*=0.015), AKI (OR=3.300; *p*=0.028), and cardiac complications (OR=3.256; *p*=0.030) were significantly associated with mortality. Our study demonstrated that the mean age of patients with NPSLE was 32.1±12.1 years (range 12-53 years), which was not significantly different from the age of patients with SLE. However, there was significant difference in mortality between age at onset of NPSLE >14 and <14 years.

### Pathophysiology of NPSLE

The pathogenic etiologies of NPSLE are likely to be multifactorial and several pathophysiological mechanisms have been implicated. NPSLE symptoms develop from injury to the vasculature, blood-brain barrier (BBB), and brain parenchyma. Studies have shown that cytokines and autoantibodies may mediate the damage, leading to focal or diffuse effects on the CNS. The PNS is not protected by a BBB, so it is susceptible to the effects of immune complexes, circulating autoantibodies, and other inflammatory molecules 10.

The brain parenchyma may be the target of infiltrating cells, cytokines, and autoantibodies, leading to either focal or diffuse injury. Cytokines and chemokines enter into the CNS through a permeabilized BBB from the peripheral circulation, or be produced within the CNS by microglia and astrocytes. Cytokines have direct effects on neurons and endothelial cells, leading to apoptosis and dysfunction. In mice, proinflammatory cytokines are associated with anhedonia, depression, lethargy, and social isolation, similar associations exist in humans 11. The cerebrospinal fluid (CSF) examination of patients with NPSLE showed that there were many proinflammatory cytokines, including a proliferation-inducing ligand (APRIL), tumor necrosis factor (TNF), interleukin-6 (IL-6), IL-1, B cell–activating factor (BAFF), and interferon alpha (IFN-α). Although these molecules can trigger inflammatory responses, the pathophysiologic mechanism(s) still needs to be further studied.

Several antibodies are likely to be involved in the pathogenesis of NPSLE. Anti-neuronal antibodies were the first autoantibodies studied and recognized for the potential pathophysiological effects of NPSLE. APL antibodies are associated with cognitive impairment and cerebrovascular disease in patients with SLE. Various effects on coagulation proteins, platelets, and endothelial cells, including up regulation of tissue factor, are attributed to APL antibodies, which are not only serological markers, but also potentially direct lead to thrombosis and other NPSLE manifestations. Alpha-tubulin is considered as a target autoantigen of SLE, especially in patients with NPSLE with severe CNS manifestations. Studies have shown that antibodies to the N-methyl-D-aspartate receptor (NMDAR) may play a pathophysiological role in emotional and cognitive impairment of SLE. Anti-glyceraldehyde 3-phosphate dehydrogenase (GAPDH) antibodies are considered as new marker impairing cognitive processes and affecting emotional changes. These autoantibodies lead to selective impairment of cognition and spatial memory, possibly due to a substantial reduction in the spines of hippocampal pyramidal cells and dendritic processes, or as a result of microglia activation 12.

The BBB protects the brain parenchyma, and its disruption causes potentially toxic cells and molecules access to the brain. Insults such as lipopolysaccharide (LPS) and systemic infections release soluble molecules, including IL-1, IL-6, and TNF, which activate brain endothelial cells, leading to up regulation of cell adhesion molecules, in turn causing BBB disruption 13. Complement activation is another factor impairing BBB integrity 14. The autoantibody must enter into brain tissue through a breach in the BBB, leading to clinical symptoms, and the location of the breach depends on the influencing factors. Vascular injury is very common in patients with SLE. Vascular abnormalities caused by vasculopathy, atherosclerotic disease, and hypercoagulability are other mechanisms leading to the pathogenesis of NPSLE. Microvascular damage leading to ischemia may result in ischemic patchy multiple sclerosis–like demyelination and cortical atrophy observed in lupus brains. Hemorrhage, tissue infarction, or more limited focal neuron damaged are caused by impaired blood flow.

### Clinical Manifestations of NPSLE

In our study, 408 NPSLE patients had 652 NP events of which 91.2% affected the CNS and 8.8% affected the PNS. Of those with NP manifestations, 81.5% were diffuse and 18.5% were focal manifestations. Most patients, 61.2% had one neuropsychiatric event, while 38.7% of the patients had two or more manifestations. In 22.8%, nervous system involvement was the initial presentation of SLE, while 57.9% of NPSLE occurred at the onset of the disease or within the first five years after the onset of SLE.

Our study, using the ACR definitions, detected the presence of 13 of the 19 syndromes; cognitive dysfunction occurred in 42.1%, headache in 31.2%, acute confusional disorder in 16.8%, cerebrovascular disease in 12.3%, mood disorder in 10.8%, seizures in 8.9%, anxiety in 6.7%, psychoses in 6.5%, movement disorders in 2.3%, and PNS impairment in 8.8%. The most common clinical manifestations were cognitive dysfunction, followed by headache and acute confusional disorder. More than 40% of SLE patients had subjective complaints of cognitive impairment. Headaches are common in patients with NPSLE, tension headaches and migraine account for the majority. Ischemic strokes occurred in 78.4% of the observed cardiovascular disease (CVD), and 54.6% of stroke patients were found to have a recurrent stroke, leading to 58.7% morbidity and a 26.8% mortality rate. Our study has identified several risk factors for stroke in patients with NPSLE, including hypertension, advanced age, cigarette smoking, previous TIA or stroke, diabetes mellitus, dyslipidemia, cardiac valvular disease, APL antibodies, and a systemic lupus erythematosus disease activity Index (SLEDAI) score of >10. Peripheral nerve involvement occurs in 8.8% of patients with SLE, symptoms can be subtle or severe and overlooked by the rheumatologists. The most common manifestation is distal sensory or motor neuropathy (65.9%). A very important finding in our study that deserves emphasis was that the occurrence of neuropsychiatric events in these patients was associated with increased organ damage and reduced quality of life. This finding reinforces the need for further studies to assess the relationship between SLE-related morbidity and mortality and NPSLE manifestations.

### Diagnostic challenges

NPSLE often presents as a diagnostic challenge, hampering early diagnostic of NPSLE is the lack of diagnostic criteria. After excluding secondary causes, NPSLE can be diagnosed if patients' NP signs and symptoms are confirmed with abnormalities in the CSF analysis, EEG, neuropsychological examination, biopsy, and neuroimaging studies 15. A careful and detailed physical examination and medical history, including a complete mental status and neurologic evaluation, and medication review, must be performed on each patient. Although NPSLE may be the sole or initial manifestation of SLE activity, our study shows that NPSLE frequently occurs when SLE is serologically and clinically active. In our study, higher SLEDAI score and APL antibody positivity increased the risk of development of NPSLE. Once NPSLE is suspected, certain antibodies may be useful for diagnosis, including APL, anti-ribosomal-P, anti-neuronal and anti-ganglioside antibodies 16,17. By combining serological and clinical analysis with imaging findings, we can determine that the patient's clinical manifestations are associated with active NPSLE rather than other causes. In our study, five signs (manifestations, disease activity, antibodies, thrombosis, and skin lesions) showed negative and positive predictive values were more than 70%, and consequently were included in the diagnostic criteria (Table 1).

To facilitate the diagnostic to the neurologic manifestations of suspected NPSLE origin and to assist the rheumatologist diagnosis it correctly, we suggest a revised set of diagnostic criteria. Our goal was to combine the current research progress with the need to simplify the diagnostic methods. As there is no diagnostic test for NPSLE, the diagnostic is confirmed by use of clinical assessment, disease activity, antibodies, thrombosis, and skin lesions. Therefore, an attempt was made to develop the diagnostic criteria for NPSLE based on cases described in the survey and literature. A confirmed diagnosis of SLE is essential for all the author's criteria for NPSLE. The diagnostic criteria proposed here are based on an evidence-based approach; however, this is merely one attempt to simplify the diagnostic path of NPSLE (Table 2). There may be a need to expand the diagnostic criteria for NPSLE, which would be achieved only through further studies.

**Figure 2:**
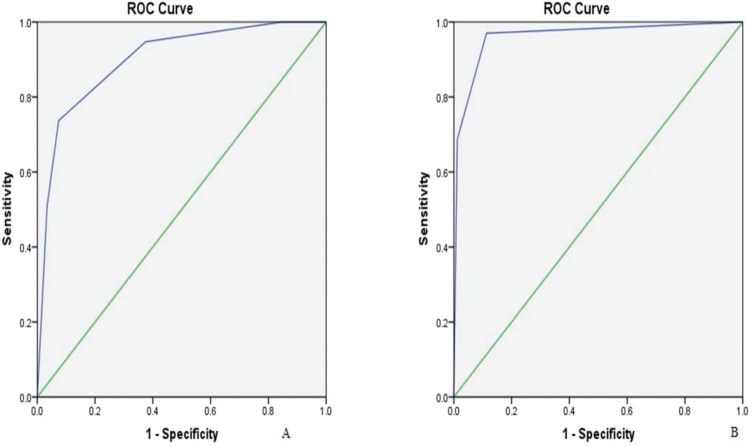
The receiver operating characteristic curve of (A) American College of Rheumatology diagnostic criteria (area under the curve=0.900) and (B) revised diagnostic criteria (area under the curve=0.962).

Since the publication of the ACR criteria, several researchers have used these criteria in their studies. The ACR criteria 18 had a high sensitivity (91%) but a low specificity (46%). Our result showed that the specificity, accuracy, and positive predictive value (PPV) of the revised diagnostic criteria were significantly higher than those of the ACR criteria (χ2=13.642, 15.591, 65.010, *p*<0.001) (Table 3). The receiver operating characteristic curve (ROC) of the revised diagnostic criteria and ACR criteria analysis were individually plotted, and their area under the curve (AUC) were calculated (Figure 2). Result showed that the AUC for the revised diagnostic criteria was 0.962 (standard error=0.015, 95% confidence intervals [CI] =0.933–0.990). The AUC for the ACR criteria was 0.900 (standard error =0.024, 95% CI=0.853–0.946). The AUC for the revised diagnostic criteria was different from that for the ACR criteria (Z=2.19, *p*<0.05). The diagnostic criteria proposed here can comprise a simplified, reliable, and cost-effective approach for diagnosing NPSLE. Our study revealed that revised diagnostic criteria had significantly better accuracy, specificity and PPV than the ACR criteria. Therefore, the revised diagnostic criteria may improve accurate diagnosis of NPSLE compared to the ACR criteria, which is reflected in the improved higher AUC, specificity, and accuracy in our study.

### Severity and prognosis of NPSLE

The prognosis of patients with major NPSLE manifestations is less optimistic 19. Coma, stroke, and status epilepticus are poor prognostic signs; aggressive evaluation and treatment are needed to prevent death or residual neurologic damage. Taking the factors including age, NP manifestations, hypertension, AKI, infection and treatments into consideration, univariate analysis showed that age <14 years at onset of NPSLE (*p*=0.001), AKI (*p*<0.001), infection (*p*=0.036), intrathecal methotrexate (MTX) plus dexamethasone (DXM) treatment (*p*<0.001) were the correlative factors of mortality. Multivariate logistic regression confirmed that infection (odds ratios [OR]=2.606, *p*=0.001), AKI (OR=4.711, *p*=0.003), and age <14 years at onset of NPSLE (OR=10.434, *p*=0.001) were risk factors for mortality, while intrathecal MTX plus DXM treatment (OR=0.12, *p*=0.006) was a protective factor. Moreover, we found that the proportion of patients under 14 years old in the deaths was significantly higher than that in the survivals (34.8% *vs*. 10.2%).

Our study indicated that the mortality for NPSLE was 11.2%, which is similar to 10.8% reported in patients with NPSLE 20. In our study, the mortality of patients with NPSLE is high, so early diagnosis and appropriate treatment are needed to improve the prognosis of NPSLE. This study sought to determine the severity of illness in patients with NPSLE, or the perceived severity, based on two indexes: the British Isles lupus assessment group (BILAG) and SLEDAI. In our study, the means±SD for BILAG and SLEDAI scores were 26.62±6.02 and 15.75±3.20, respectively. Patients who died had significantly higher scores than those who survived: died *versus* survived=27.25±5.34 *versus* 16.88±3.87 (*p*=0.001) for BILAG and 21.25±2.19 *versus* 18.13±2.53 (*p*=0.019) for SLEDAI, respectively.

### Management and treatment challenges

The management of patients with NPSLE includes providing symptomatic treatment, correcting the aggravating factors, and taking specific measures related to the disease process. Clinicians should try to identify and correct the possible aggravating factors, including metabolic or blood pressure abnormalities, and possible offending drugs. Therapy should not be delayed until the test results are available. Symptomatic treatment will be required according to the nature of NPSLE, and it may be initiated before disease-specific treatment. Patients' manifestations such as headaches, anxiety or an infrequent seizure, or dysphoria, paresthesias may only need psychotropic medications, analgesics and antiseizure medications or neuroleptic agents, psychological support, respectively, and closely observe the progress of the nervous system 18.

The treatment of NPSLE is mainly empirical, because few controlled clinical trials have been conducted. For patients with NPSLE who have progressive or severe, nonthrombotic or diffuse symptoms such as severe depression, acute confusional state, coma, psychosis, and aseptic meningitis, immunosuppressive therapy is beneficial in addition to symptomatic treatment. Most rheumatologists recommend taking 1 mg/kg prednisone daily in divided doses 21. Conversion of prednisone to DXM (once a day, 12 to 20 mg) is another alternative, which is more effective than other corticosteroids in crossing the BBB. Continued failure to respond is an indication of plasmapheresis or cytotoxic medications or both, especially for comatose patients. In patients who did not respond to corticosteroid therapy, intravenous cyclophosphamide (CYC, 0.75 to 1.0 g/m^2^) given every 3 to 6 weeks has been reported to be beneficial 22,23. Intrathecal MTX plus DXM (10 mg each time, once a week for 3 weeks) has been used successfully in our study, which has a rapid onset of action, less side effects, can improve drug bioavailability and avoid systemic toxicity, is an effective treatment for NPSLE (Table 4).

Patients with NPSLE with thrombotic or focal manifestations need to be evaluated immediately and aggressively. If vasculitis is suspected, high doses of corticosteroids similar to patients with nonthrombotic, diffuse, or severe manifestations are used. Cytotoxic medications should be used as early as possible. Clinical studies have shown that CYC is more effective than other immunosuppressive agents. Plasmapheresis may be beneficial within the first week to allow time for the CYC and corticosteroids to take effect 21. Whether long-term antiplatelet therapy can prevent atheroma formation or thrombosis in the damaged vessel is unclear, but it is often used clinically 24.

Most strokes caused by thrombosis are associated with APL antibodies, while others are caused by emboli from damaged heart valves 25. These patients should be treated with antiplatelet drugs, statins, hydroxychloroquine, and/or anticoagulation therapy. Many experts recommend that warfarin be used lifelong in patients with cerebral artery thrombosis, maintaining the international standardized ratio (INR) of 3.0 to 3.5 21. In addition, any patient with a lupus anticoagulant and recurrent strokes should undergo testing for chromogenic factor X levels and periodic factor II; both these values should be maintained at 15% to 20% of normal to ensure adequate anticoagulation. Studies suggest that antimalarial agents (AMs) may prevent CNS flares, protect against worsening of brain lesions protect from seizures, but not from headaches 23,26,27. In addition, AMs diminish the risk for thrombosis by reducing blood viscosity, red blood cell sludging, and platelet aggregation; all of these can lead to cerebrovascular diseases in the context of APL antibodies 28.

Patients with moderate to high titers of APL antibodies, cerebral infarctions, and seizures should be started on anticoagulation therapy once seizures are controlled, despite the higher risk of falls and cerebral trauma. In steroid-unresponsive NPSLE, uncontrolled trials showed that B cell depletion therapy with anti-CD20 (rituximab) was effective 29. High-dose CYC therapy or hematopoietic stem cell transplantation may be considered for patients with resistant and severe NPSLE 21. Patients with chronic inflammatory demyelinating polyneuropathy (CIDP) or Guillain-Barré syndrome frequently undergo plasmapheresis or intravenous immunoglobulin as additional therapy. Patients with mononeuritis multiplex due to vasculitis should also be treated with cytotoxic medications such as CYC. In severe cases such as transverse myelitis, early combination of glucocorticoid and CYC can provide better outcomes than glucocorticoid alone 23.

### Future Directions

With the further study of the mechanism of immune abnormalities associated with active SLE, new therapies are being developed and new targets have been identified for the treatment of active disease. However, no new potential agents are on the horizon for NPSLE. Understanding the pathophysiologic mechanisms leading to NPSLE is essential for the evaluation and design of effective interventions 30. Several difficult clinical situations need to be further elucidated. First, we currently lack comprehensive understanding of the mechanisms involved in NPSLE. Second, diagnostic tools and criteria for patients with NPSLE are limited because there are no *in vivo* imaging biomarkers providing direct evidence for BBB dysfunction 31. Third, randomized control trials (RCTs) to evaluate specific treatments for NPSLE are limited, and treatment strategies are based on small control trials and expert recommendations. There are few studies on NPSLE treatment; running such studies is difficult, because each trial may require a large number of patients and collaboration between centers from several countries. Finally, in clinical settings, the diagnosis and treatment of NPSLE requires the collaboration of neurologists and psychologists. In the next 10 years, the combination of NPSLE pathophysiological mechanisms, RCTs, and new techniques and methods may be helpful for the individualized treatment of patients with NPSLE. The clinical significance of symptom combination patterns and each symptom needs to be further studied.

## AUTHOR CONTRIBUTIONS

Zhang Y designed the study protocol and drafted the manuscript. Han H and Chu L analyzed data and checked the manuscript. All authors have read and approved the content of the manuscript.

## Figures and Tables

**Table 1:** Sensitivity and specificity of the symptoms and signs of neuropsychiatric lupus erythematosus and analysis of their positive and negative predictive values.

Symptoms and signs	Sensitivity (%)	Specificity (%)	Positive predictive value (%)	Negative predictive value (%)
Manifestations^a^	96.4	37.1	70.6	86.5
Higher SLEDAI score	80.6	42.1	79.4	73.9
Antibody positive^b^	84.0	53.1	71.1	71.8
Thrombosis	78.0	63.2	70.8	71.4
Skin lesions	60.0	96.3	94.2	70.7

a Altered cognitive and/or neuropsychiatric status manifestations or sensorimotor neuropathy.

b Antiphospholipid (APL), anti-ribosomal-P (ARP), anti-neuronal, anti-ganglioside (GMI) and anti-Ro antibody, one of the antibodies is positive.

**Table 2:** Definition and diagnostic criteria for neuropsychiatric lupus erythematosus.

**Definition of NPSLE** NPSLE is a group of neurologic syndromes of the central and peripheral nervous system and/or of psychiatric syndromes observed in patients with SLE in which other causes are excluded by laboratory, clinical, neuropsychological tests and neuroimaging.
**Prerequisite for diagnosis:** All the author’s criteria for NPSLE require the diagnosis of SLE. Symptoms and signs: 1. Altered cognitive and/or neuropsychiatric status manifestations or sensorimotor neuropathy. 2. Higher SLEDAI score (>10). 3. Antiphospholipid (APL), anti-ribosomal-P (ARP), anti-neuronal, anti-ganglioside (GMI) and anti-Ro antibody, one of the antibodies is positive. 4. Complicated with tissue infarction, hemorrhage, or more limited focal neuron injury results from impaired blood low (thrombosis). 5. Skin lesions
**Diagnosis** **Grade of NPSLE combinations of features requirements for diagnosis** NPSLE1: SLE plus at least one altered cognitive and/or neuropsychiatric status manifestations or sensorimotor neuropathy and one of the following: higher SLEDAI, antibody positivity, thromboembolism, or skin lesions. NPSLE2: SLE and at least one altered cognitive and/or neuropsychiatric status manifestations or sensorimotor neuropathy.
**Exclusion and provisions** Secondary causes such as medication side effects (especially steroids), thyroid disease, infections, metabolic disturbances, TTP, valvular heart disease, depression, sleep apnea, and psychosocial- or functional-related conditions need to be excluded.

NPSLE 1: ‘‘definite’’ NPSLE; NPSLE 2: ‘‘suspected’’ NPSLE. TTP: thrombotic thrombocytopenic purpura.

**Table 3:** Comparison of the diagnostic performance of the two diagnostic criteria.

Examination method	Sensitivity (%)	Specificity (%)	Accuracy (%)	Positive predictive value (%)	Negative predictive value (%)
ACR criteria	87.7	44.4	68.6	66.7	74.1
Revised criteria	82.8	71.4	80.9	93.3	46.3
χ^2^	2.151	13.642	15.591	65.010	16.248
*p*	0.142	<0.001	<0.001	<0.001	<0.001

**Table 4:** Comparison of the intravenous cyclophosphamide and intrathecal methotrexate plus dexamethasone.

Treatment	Number of patients	Response duration (days)	Effective rate (%)	Incidence of adverse events (%)
CYC	72	5.2±1.03	72.2	17.3
MTX+DXM	84	2.3±0.95	90.5	6.0
χ^2^ or t	-	6.539	4.386	5.112
*p*	-	<0.001	0.036	0.024
